# Automated CT detection of intestinal abnormalities and ischemia for decision making in emergency medicine

**DOI:** 10.1186/1475-925X-13-S1-S3

**Published:** 2014-02-28

**Authors:** Taichiro Tsunoyama, Tuan D Pham, Takashi Fujita, Tetsuya Sakamoto

**Affiliations:** 1 School of Medicine, Department of Emergency Medicine, Trauma and Resuscitation Center, Teikyo University, Tokyo 173-8606, Japan; 2 Aizu Research Cluster for Medical Engineering and Informatics, Research Center for Advanced Information Science and Technology, The University of Aizu, Aizuwakamatsu, Fukushima 965-8580, Japan

## Abstract

**Background:**

Evaluation of computed tomography (CT) for the diagnosis of intestinal wall abnormalities and ischemia is important for clinical decision making in patients with acute abdominal pain to which if surgery should be performed in the emergency department. Interpretation of such information on CT is usually based on visual assessment by medical professionals and still remains a challenge in a variety of settings of the medical emergency care. This paper reports a pilot study in the implementation of image processing methods for automated detection of intestinal wall abnormalities and bowel ischemia, which can be of a potential application for CT-based detection of the intestinal disease.

**Methods:**

CT scans of 3 patients of ischemia, one benign and one control subjects were used in this study. Statistical and geometrical features of the CT scans were extracted for pattern classification using two distance measures and the *k*-nearest neighbor algorithm. The automated detection of intestinal abnormalities and ischemia was carried out using labeled data from the training process with various proportions of training and testing samples to validate the results.

**Results:**

Detection rates of intestinal ischemia and abnormalities are promising in terms of sensitivity and specificity, where the sensitivity is higher than the specificity in all test cases. The overall classification accuracy between the diseased and control subjects can be as high as 100% when all CT scans were included for measuring the difference between a cohort of three patients of ischemia and a single control subject.

**Conclusion:**

The proposed approach can be utilized as a computer-aided tool for decision making in the emergency department, where the availability of expert knowledge of the radiologist and surgeon about this complex bowel disease is limited.

## Introduction

Intestinal or bowel ischemia is a complex disease caused by various conditions that result in the interruption or loss of the blood supply of the intestines [1]. With the increase in average life expectancy, acute bowel ischemia represents one of the most threatening abdominal conditions in elderly patients [2]. In particular, the lack of blood flow to the small intestine can be due to thromboembolism, nonocclusive causes, bowel obstruction, neoplasms, vasculitis, abdominal inflammatory conditions, trauma, chemotherapy, radiation, and corrosive injury [3,4]. It has been reported that computed tomography (CT) can reveal changes in image signals because of ischemic bowel and therefore important clinical data for the effective diagnosis of the primary cause of ischemic bowel disease [3]. Furthermore, CT is increasingly being used as a tool for evaluation of patients with acute symptoms of intestinal disease because of its wide availability and assessment of other extraintestinal abnormalities.

As another aspect of the disease, there is a demand for the detection of small-bowel ischemia associated with small-bowel obstruction, which is a frequent clinical situation in the emergency department [5,6]. CT again can reveal the site, level, cause, and severity of obstruction and to display the presence of strangulation and signs of threatened bowel viability; in case of radiation inflammation, CT provides useful information about small-bowel damage [7]. However, common CT findings in acute small bowel ischemia are not specific and, therefore, it is often a combination of clinical, laboratory and radiologic signs that may lead to a correct diagnosis. The reliable recognition of ischemic small bowel disease with the spectrum of diagnostic CT signs is currently a challenge for both the radiologist and the surgeon, particularly in case of emergency medicine [3,5].

Although CT findings have been found to be useful for the detection of small-bowel ischemia and bowel-wall abnormalities, challenges in diagnostic performance of CT impose difficulties in the assessment of emergency department patients in terms of sensitivity (the true positive rate that indicates the percentage of patients who are correctly identified as having the disease) and experts' disagreements [5]. In particular, radiologists find it difficult to detect intestinal ischemia associated with small-bowel obstruction because the bowel lesion of intestinal ischemia is usually thin caused by luminal expansion [6]. In patients with intestinal strangulation obstruction, if surgeries are delayed for more than 36 hours after the sympton onset due to the inability of ischemia detection, mortality can be as high as 25% [6]. When oxygen is inadequately supplied to intestines, ischemia can affect the small intestine, leading to acute and chronic pain. This condition is known as mesenteric ischemia and characterized with high mortality [8]. Major progresses in imaging technology, in which CT angiography is the current gold standard, for diagnostic and treatment of acute mesenteric ischemia have been reported over the past decades [9-11]. However, mortality caused by acute mesenteric ischemia still remains high, therefore early detection of the symptom of this disease is a challenging task for physicians [9].

Current evaluation of CT in the detection of intestinal ischemia and obnormalities is done by visual assessment, and the inspection by measuring the attenuation of the regions of interest of the bowel wall in either contrast-enhanced or unenhanced CT scans is a novel study recently reported [6]. However, such measurement of the region of interest is manually performed at selected obstructed small-bowel loops on the CT scans. Another manual image analysis approach for CT diagnosis of abnormal bowel wall is by selecting CT attentuation patterns, including white, gray, water halo sign, fat halo sign, and black [12].

In general, there have been rarely any efforts toward the development of automated image analysis for detecting intestinal ischemia and the abnormal bowel wall. Therefore, the motivation of this research is to investigate the feasibility of building an automated image analysis system by the incorporation of clinical knowledge with image processing and pattern recognition methods. The proposed automated system is expected to assist general surgeons to quickly identify acute small-bowel ischemia and intestinal abnormalities from CT information about emergency department patients with abdominal pain. Such a computer-aided tool will be very useful in the urgent diagnosis of this complex disease and greatly benefit the patient. The research presented in this paper is original because it is the first of its type in the automated image analysis of CT scans of the complex disorder.

## Materials and methods

### CT data

CT scans were obtained from patients who had surgery because of the peritonitis with the suspicion of the bowel ischemia except for one control patient who was admitted in our unit for other reason. The patients were diagnosed by physical examination, blood tests and CT scans. Important physical findings of peritonitis were the muscular defense and rebound tenderness. The lactic acidosis elevation suggested the bowel ischemia in the blood test. If the renal function of the patient was normal, intravenous contrast (IV) was used to enhance the CT. The CT finding was read by the attending radiologist. The CT patterns of the black and gray attenuation in the intestine were indicators of bowel ischemia. After the diagnosis of peritonitis with bowel ischemia, the emergency surgery was carried out. If the bowel was necrotic, it was resected.

**Figure 1:**
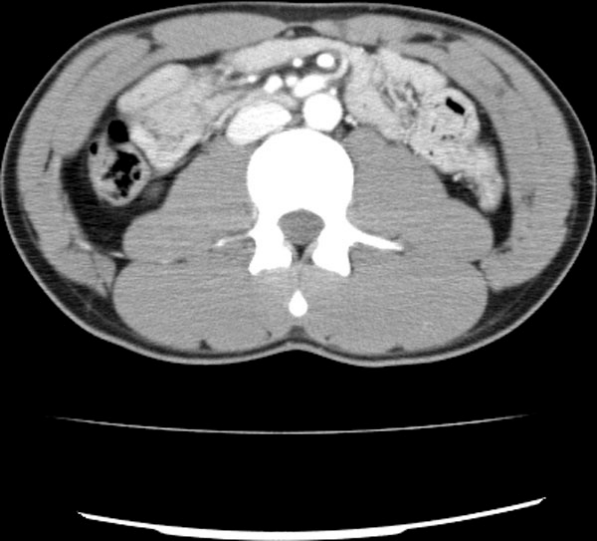
**Axial CT scan obtained with IV contrast material in 17-year-old male control subject**.

**Figure 2:**
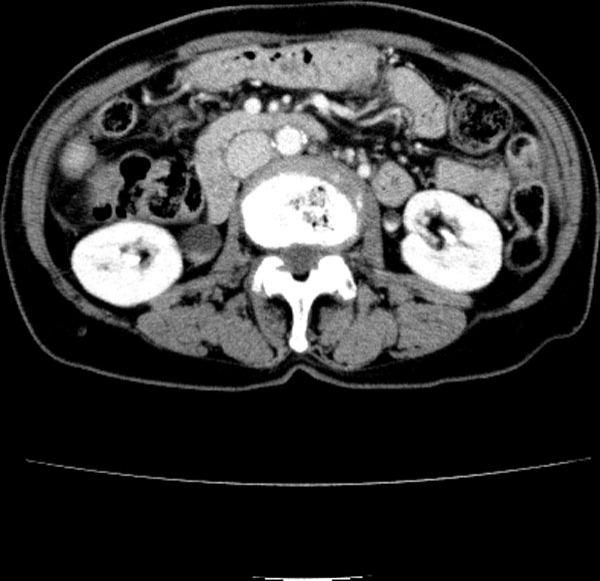
**Axial CT scan obtained with IV contrast material in 76-year-old female benign subject**.

**Figure 3:**
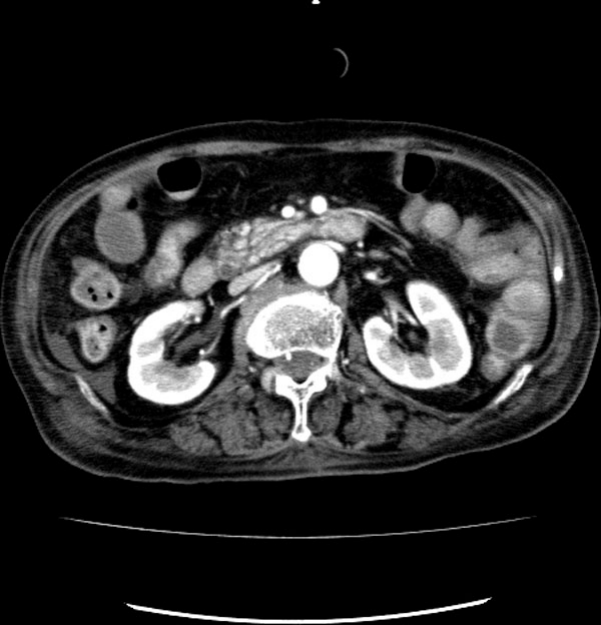
**Axial CT scan obtained with IV contrast material in 80-year-old female subject diagnosed with ischemia**.

**Figure 4:**
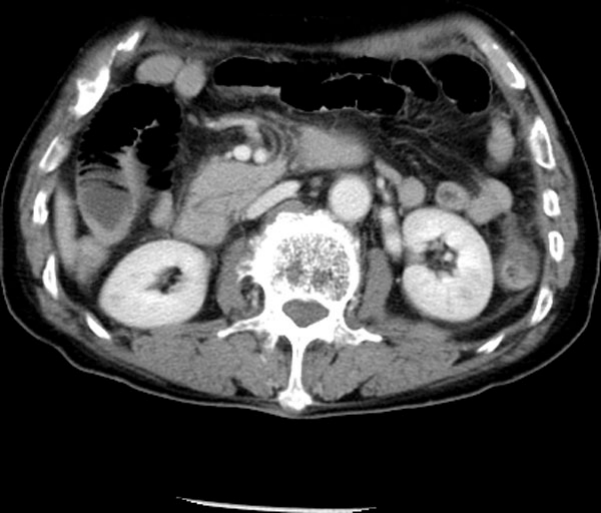
**Axial CT scan obtained with IV contrast material in 76-year-old male subject diagnosed with ischemia**.

**Figure 5:**
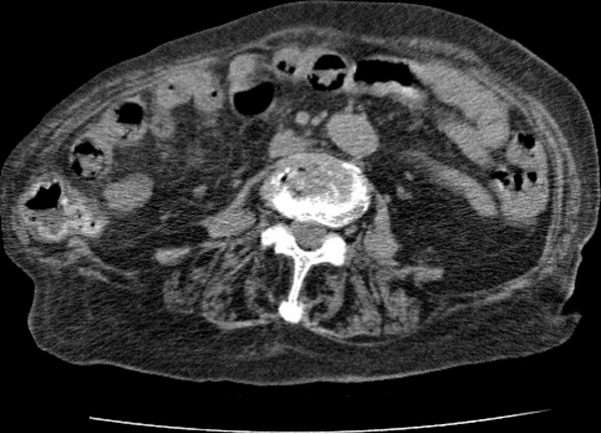
**Axial CT scan obtained with IV contrast material in 84-year-old female subject diagnosed with ischemia**.

Five subjects were included in this study: three patients of ischemia, one benign and one control. The three ischemic patients had surgery. The ischemic bowel was resected in three patients. One patient had surgery without the bowel resection. One 17-year-old male patient is the control subject who had his whole body CT scanned due to other injury and his abdominal CT was normal. In the ischemic cohort, the 80-year-old (Patient 1) and 87-year-old (Patient 2) female patients had small-bowel resection because of small-bowel ischemia, and the 76-year-old male (Patient 3) had his descending colon resected because of colon ischemia. The benign subject is the 76-year-old female who underwent surgery without bowel resection. The CT and operation findings of the subjects were compared. This retrospective study was approved by the patients' informed consents, which waived approval by our institutional review board. Figures 1, 2, 3, 4, 5 show typical intravenous contrast-enhanced CT scans of the control, benign and three patients of ischemia; respectively.

### Clinical feature extraction

To utilize the medical knowledge about the CT diagnosis of intestinal abnormalities and ischemia, which can be used for computerized extraction of features of the regions of interests in the CT images; five attenuation patterns described in [12], which has previously been discussed, are adopted in this study to train computer methods for automated detection of the disease. These patterns include *white*, *gray*, *water halo sign*, *fat halo sign*, and *black *[12].

The interpretations of these five attenuation patterns are as follows [12]. The white attenuation pattern describes the contrast material enhancement affecting the thickened bowel wall. The gray attenuation pattern is the sign of a thickened bowel wall with limited enhancement. This pattern of gray attenutaion is often used to differentiate between benign and malignant disease. The water halo sign indicates stratification within a thickened bowel wall that consists of thickened layers. The fat halo sign presents a three-layered target sign of thickened bowel in which the middle layer has a fatty attenuation, referring to Crohn disease in the small intestine and inflammatory bowel diseases in the colon. The black attenuation pattern, which particularly shows air trapped between the bowel wall, indicates ischemia.

### Computerized feature extraction

CT patterns that are similar to the clinical features, which have been previously presented, are extracted using computational methods and applied in this study for classifying diseased, benign and control subjects. Basic concepts and mathematical formulations of the computerized features are briefly presented in this section.

#### Summary statistics

The image histogram provides information about the distribution of the intensity that can be captured by summary statistics and utilized for feature extraction of images. The summary statistics are divided into three categories: measures of location, measures of spread and measures of shape [13]. The statistics in the first category involves the mean, median and mode, which give information about where various components of the gray-level distribution locate. The second statitical category includes the variance and standard deviation to describe the variability of the gray levels. The third category employs the coefficients of skewness and kurtosis to quantify the shape of the gray-level distribution of the image histogram, relative to a normal distribution that has skewness and kurtosis of 0. These three types of summary statistics; including the mean, variance, skewness (measure of histogram asymmetry about the mean) and kurtosis (measure of histogram sharpness) [14], will be applied for extracting discriminative information from the CT patterns. Let *N *be the the total number of pixels and *x_i _*the gray level of pixel *i*, the intensity mean, *m*, is the arithmetic average intensity is defined as

(1) $$m=\frac{1}{n}\overset{N}{\underset{i=1}{\sum }}x_{i}.$$

The intensity variance, *σ*^2^, is given by:

(2) $$\sigma^{2}=\frac{1}{N}\overset{N}{\underset{i=1}{\sum }}{\left(x_{i}-m\right)}^{2}$$

where the square root of the variance, *σ*, is the standard deviation.

The coefficient of skewness, *g*_1_, can be calculated as follows:

(3) $$g_{1}=\frac{\frac{1}{N}\sum_{i=1}^{N}{\left(x_{i}-m\right)}^{3}}{\sigma^{3}}.$$

The coefficient of kurtosis, *g*_2_, is given by

(4) $$g_{2}=\frac{\frac{1}{N}\sum_{i=1}^{N}{\left(x_{i}-m\right)}^{4}}{\sigma^{4}},$$

which is computed almost the same way as the coefficient of skewness by replacing the exponent 3 with 4.

#### Gray-level co-occurrence matrix

A gray-level co-occurrence matrix (GLCM) or co-occurrence distribution of an image is a matrix or distribution that is defined over an image to be the distribution of co-occurring values at a given offset intensity value [15]. In other words, the GLCM contains elements that are counts of the number of pixel pairs for certain intensity levels, when separated by a distance *θ*(*d_r_*, *d_c_*), where *d_r _*and *d_c _*are the row-wise and column-wise positions, respectively. Given  as a set of image intensity levels, p,q∈L, and denote *θ*(*d_r_*, *d_c_*) as *θ *for short; an element of the GLCM of size *L *× *L*, where *L *is the cardinality of , can be mathematically expressed as

(5) $$c_{pq}=\overset{N\left(\theta \right)}{\underset{\left(u,v\right)|d\left(u,v\right)=\theta }{\sum }}\left(x_{u}=p\right)\wedge \left(x_{v}=q\right)$$

where *x_u _*and *x_v _*are the gray levels of the two pixels at locations *u *and *v*, respectively; *u *and *v *are separated by the distance *d*(*u*, *v*); ∧ stands for the logical AND operator; and *N*(*θ*) is the total number of pairs of *x_u _*= *p *and *x_v _*= *q *offset by *θ*.

The probability of a GLCM element can be defined by

(6) $$a_{pq}=\frac{c_{pq}}{\sum_{p=1}^{L}\sum_{q=1}^{L}c_{pq}}$$

based on which many descriptors can be developed such as maximum probability, correlation, contrast, energy or uniformity, homogeneity, and entropy [16,17]. Here the GLCM entropy is utilized to consider the uncertainty information of the image to complement the features of summary statistics. Let *A *be the image spatial distribution associated with the co-occurrence matrix, its entropy is computed, using the basic information theory, as

(7) $$H\left(A\right)=-\overset{L}{\underset{p}{\sum }}\overset{L}{\underset{q}{\sum }}a_{pq}\log a_{pq}$$

where log(0) = 0.

#### Fractal dimension

One of the most popular methods for calculating fractal dimensions (FD) is the box-counting method [18]. The fractal dimension of images have been used to characterize texture and complex structures that inherently exists in medical images [19,20]. The box-counting method determines the dimension of a geometrical object by constructing *boxes *of side length *r *to cover the space occupied by the object. In one dimension, the boxes are line segments. For a two-dimensional space, the boxes are squares. In three dimensions, they are cubes, and so on. The box-counting dimension *d_B _*is defined as [21]

(8) $$n\left(r\right)=\underset{r\to 0}{\lim}kR^{-d_{B}}$$

where *n*(*r*) is the minimum number of boxes needed to contain all the points of the geometrical object, and *k *is a constant.

The box-counting dimension *d_B _*can be obtained by taking the logarithm of both sides of Equation (8), yielding

(9) $$d_{B}=\underset{r\to 0}{\lim}\left[-\frac{\log\left[n\left(r\right)\right]}{\log\left(r\right)}+\frac{\log\left(k\right)}{\log\left(r\right)}\right].$$

The last term in Equation (9) becomes negligible as *r *approaches zero, giving

(10) $$d_{B}=-\underset{r\to 0}{\lim}\frac{\log\left[n\left(r\right)\right]}{\log\left(r\right)}$$

based on which *d_B _*can be estimated by taking the negative sign of the slope of a straight line fit to the curve of log(*r*) (x-axis) vs. log[*n*(*r*)] (y-axis).

### Feature normalization

To overcome the variations of different features that may have different dynamic ranges and features with large values will have a stronger bias in the calculation of some distance measure for pattern classification. Therefore feature normalization is adopted to restrict the values of all features within a normalized range by transforming the features to zero mean and unit variance as [22]

(11) $$f^{*}=\frac{f-\mu_{f}}{\sigma_{f}}$$

where f* is the normalized feature, *f *the unnormalized feature, and *μ_f _*and *σ_f _*are the mean and standard deviation of the unnormalized feature; respectively.

### Distance measures and classification

Two types of distance measures were applied in this study to calculate the similarity between two classes for pattern classification: Euclidean and Bhattacharyya [22]. The Euclidean distance, *D_E_*, of the two feature vectors **f **= (*f*_1_, *f*_2_, ..., *f_n_*) and **q **= (*q*_1_, *q*_2_, ..., *q_n_*) is defined as

(12) $$D_{E}\left(f,q\right)=\sqrt{\overset{n}{\underset{i=1}{\sum }}{\left(f_{i}-q_{i}\right)}^{2}}.$$

The Bhattacharyya distance, *D_B_*, which is defined as [22]

(13) $$D_{B}=\frac{1}{8}{\left(m_{i}-m_{j}\right)}^{T}{\left(\frac{C_{i}+C_{j}}{2}\right)}^{-1}\left(m_{i}-m_{j}\right)+\frac{1}{2}\ln\frac{\left|\frac{C_{i}+C_{j}}{2}\right|}{\sqrt{\left|C_{i}||C_{j}\right|}}$$

where **m***_i _*and **C***_i _*are the mean vector and covariance matrix of class *i*, respectively; and |·| denotes the determinant of the respective matrix.

The basis for applying the two types of distance measures in this study is that the Euclidean distance allows the classification using each feature vector, by which detailed classification rates for a small dataset can be revealed with the presentation of a confusion matrix; whereas the Bhattacharyya distance was used as a class separability measure and can provide information about the disease detection using the collections of sample features of the diseased, benign, and control groups. Based on each of the distance measures, the classification was carried out using the *k*-nearest neighbor (*k*-NN) classifier [23], which assigns the unknown or test sample to the class that has the largest number of closest distances with the test sample.

### Searching for regions of interest

**Figure 6:**
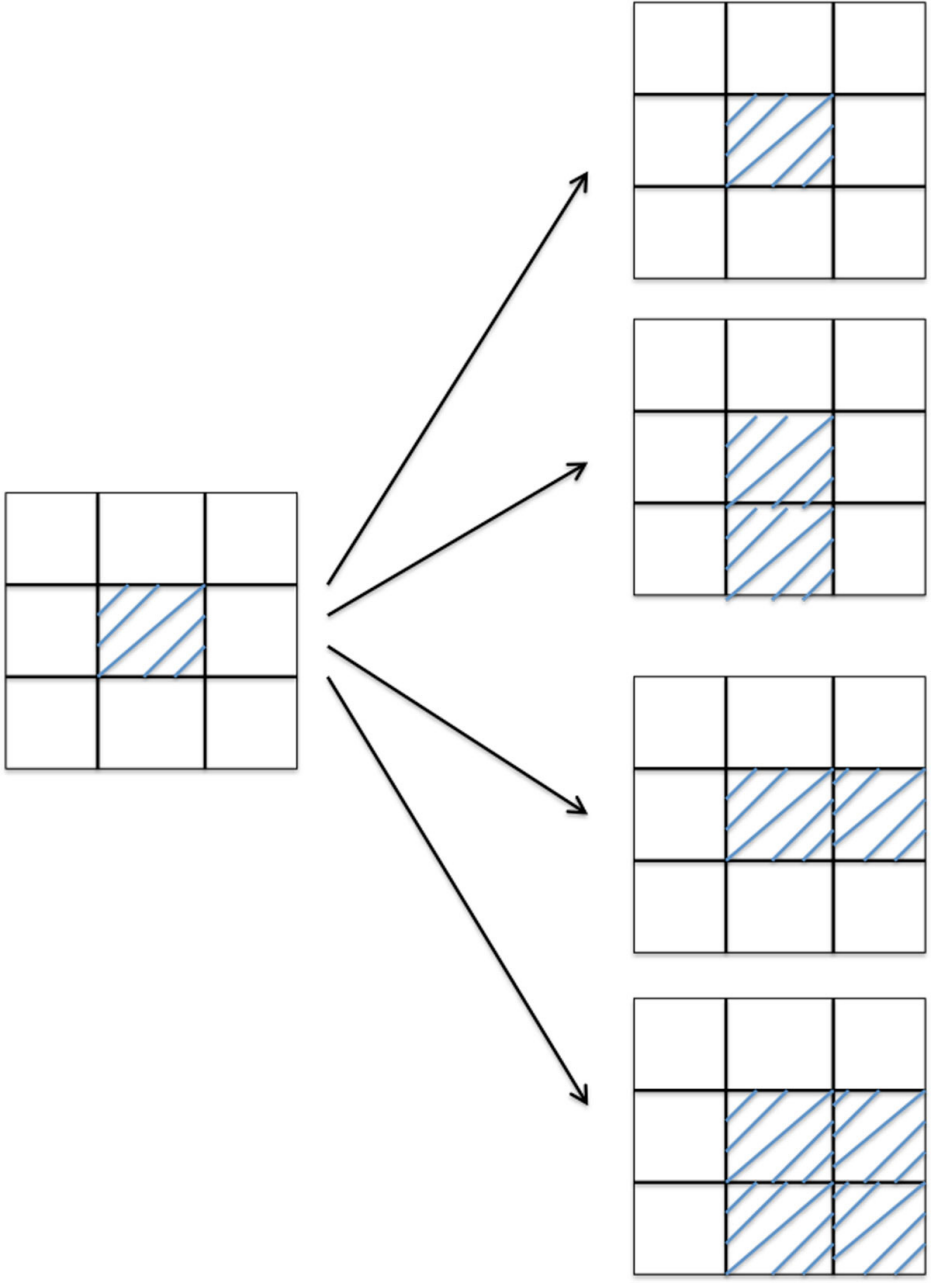
**Diagram showing a strategy for searching a potential pattern that closely matches any of the labeled patterns**. The search uses a window having the average size of the trained windows and if being similar the window moves one window down, one window left, and one window to the diaggonal direction. Any window that satisfies the criterion of similarity is retained.

In order to automate the detection of bowel abnormalities and ischemia, a computerized identification for potential regions of interest is developed by a forward search strategy. This forward search uses a window that has the average size of all the trained patterns to start at the top-left corner of the image and moves forward from left to right and from top to bottom of the image. The feature vector extracted from the sub-image occupied by the searching window is matched against the feature vector of each of the trained patterns using a Euclidean distance measure. If the similarity is greater than a certain threshold, then the window expands to the right, below, and diagonal positions (Figure 6) each of which will be retained if the similarity satisfies the threshold criterion, which is defined using the definition of the relative error as follows [24].

(14) $$\left(\frac{F-\hat{F}}{F}\right)\;\times \;100=\left(1-\frac{\hat{F}}{F}\right)\;\times \;100$$

where *F *is the value of the test feature and $\hat{F}$ the value of the labeled feature if $F>\hat{F}$; otherwise *F *is the value of the labeled feature and $\hat{F}$ the value of the test feature.

## Results

**Figure 7:**
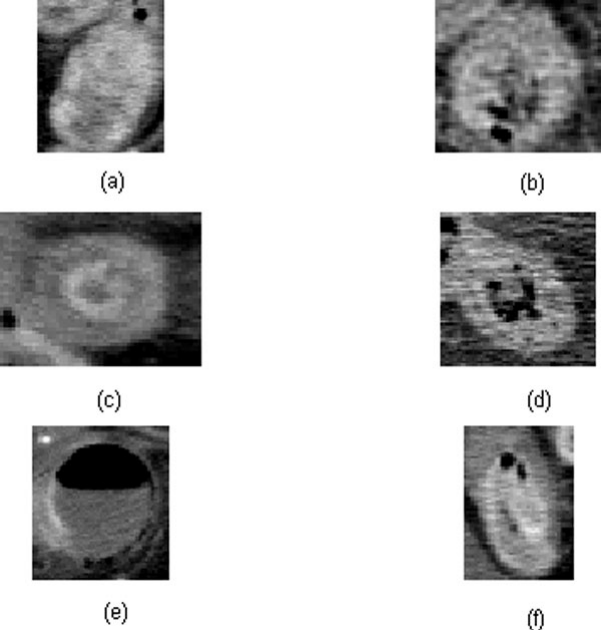
**Extracted CT patterns that are similar to (a) white, (b) gray, (c) water halo sign, (d) fat halo sign, (e) black, and f) air-trapped**.

**Figure 8:**
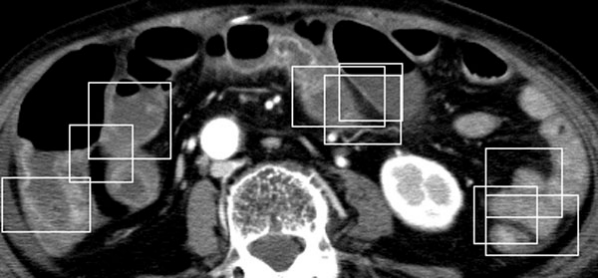
**A typical result of the automated searching for the regions of interest in a CT scan of the 80 year-old female patient having ischemia: small windows indicate detected regions of interest using the proposed forward search, thus alleviating the need for the image segmentation of the intestines**.

Both available IV contrast-enhanced and unenhanced CT scans of each subject, which contain images of the intestines, were used to test the performance of the proposed approach. The window sizes in pixels, which were selected as the average dimensions of the window sizes of all trained attenuation patterns for searching potential regions of interest are: 55 by 59 for white, 53 by 53 for gray, 42 by 46 for water halo, 59 by 54 for fat halo, 53 by 56 for black, 50 by 55 for ischemia, 45 by 48 for gray attenuation of benign, 44 by 66 for control-like intensity of benign, and 51 by 55 for control. The spatial orientation of the GLCM was selected as "one pixel to the right and one pixel down" (diagonal of 45 degrees). Figure 7 shows some extracted CT images of white, gray, water halo sign, fat halo sign, black, and air-trapped patterns of the data under study. Figure 8 shows typical results of the automated searching for the regions of interest in CT scans of the five subjects.

The data were randomly split into several proportions for training and testing the proposed approach. The spatial orientation of the GLCM was selected as "one pixel to the right and one pixel down" (diagonal of 45 degrees). Tables 1, 2, 3 are the confusion matrices using the Euclidean distance measure and *k*-NN (*k *= 3) classifier. The results shown in Table 1 were obtained by using 50% of the pattern features for training and the other 50% for testing. For Table 2, 60% of the features were used for training and the other 40% for testing. The confusion matrix shown in Table 3 was obtained with 70% of the features for training and the other 30% for testing. The leave-one-out method was used for the random selection of training and testing data to enhance the statistics of the experimental results, which are shown in Table 4. Tables 5, 6, 7 show the confusion matrices using the Bhattacharyya distance measure with (50% training, 50% testing), (50% training, 40% testing) and (70% training, 30% testing); respectively.

**Table 1:** Confusion matrix: 50% of patterns used for training and other 50% for testing, using Euclidean measure.

	White	Gray	Water	Fat	Black	Ischemia	Benign	Control
White	0.538	0.115	0.007	0.053	0.000	0.211	0.000	0.076
Gray	0.222	0.426	0.010	0.040	0.051	0.235	0.017	0.000
Water	0.130	0.257	0.080	0.060	0.240	0.230	0.003	0.000
Fat	0.075	0.052	0.035	0.785	0.023	0.030	0.000	0.000
Black	0.000	0.028	0.082	0.053	0.773	0.065	0.000	0.000
Ischemia	0.224	0.101	0.047	0.034	0.120	0.442	0.015	0.016
Benign	0.030	0.343	0.083	0.000	0.147	0.390	0.007	0.000
Control	0.123	0.003	0.000	0.000	0.000	0.056	0.000	0.818

**Table 2:** Confusion matrix: 60% of patterns used for training and other 40% for testing, using Euclidean measure.

	White	Gray	Water	Fat	Black	Ischemia	Benign	Control
White	0.532	0.113	0.009	0.047	0.000	0.235	0.000	0.064
Gray	0.217	0.418	0.020	0.040	0.043	0.241	0.021	0.000
Water	0.090	0.280	0.105	0.045	0.250	0.230	0.000	0.000
Fat	0.034	0.050	0.056	0.834	0.014	0.012	0.000	0.000
Black	0.000	0.016	0.066	0.054	0.811	0.053	0.000	0.000
Ischemia	0.195	0.098	0.066	0.027	0.136	0.447	0.022	0.010
Benign	0.025	0.345	0.075	0.000	0.190	0.345	0.020	0.000
Control	0.180	0.000	0.000	0.000	0.000	0.040	0.000	0.780

**Table 3:** Confusion matrix: 70% of patterns used for training and other 30% for testing, using Euclidean measure.

	White	Gray	Water	Fat	Black	Ischemia	Benign	Control
White	0.594	0.081	0.000	0.051	0.000	0.200	0.000	0.073
Gray	0.247	0.393	0.003	0.040	0.047	0.266	0.004	0.000
Water	0.080	0.385	0.015	0.050	0.305	0.165	0.000	0.000
Fat	0.060	0.018	0.040	0.853	0.005	0.025	0.000	0.000
Black	0.000	0.026	0.050	0.040	0.819	0.066	0.000	0.000
Ischemia	0.227	0.089	0.049	0.027	0.127	0.446	0.026	0.009
Benign	0.010	0.350	0.100	0.000	0.175	0.365	0.000	0.000
Control	0.147	0.000	0.000	0.000	0.000	0.010	0.000	0.843

**Table 4:** Confusion matrix: leave-one-out method used for testing, using Euclidean measure.

	White	Gray	Water	Fat	Black	Ischemia	Benign	Control
White	0.667	0.083	0.000	0.042	0.000	0.167	0.000	0.042
Gray	0.208	0.417	0.000	0.042	0.000	0.333	0.000	0.000
Water	0.000	0.000	0.000	0.000	0.000	0.000	0.000	1.000
Fat	0.000	0.000	0.077	0.923	0.000	0.000	0.000	0.000
Black	0.000	0.000	0.042	0.000	0.875	0.083	0.000	0.000
Ischemia	0.189	0.094	0.094	0.019	0.094	0.472	0.038	0.000
Benign	0.000	0.167	0.167	0.000	0.167	0.500	0.000	0.000
Control	0.111	0.000	0.000	0.000	0.000	0.000	0.000	0.889

**Table 5:** Confusion matrix: 50% of patterns used for training and other 50% for testing, using Bhattacharyya measure.

	Ischemia	Benign	Control
Ischemia	1	0	0
Benign	0.77	0.23	0
Control	0	0	1

**Table 6:** Confusion matrix: 60% of patterns used for training and other 40% for testing, using Bhattacharyya measure.

	Ischemia	Benign	Control
Ischemia	1	0	0
Benign	0.75	0.25	0
Control	0	0	1

**Table 7:** Confusion matrix: 70% of patterns used for training and other 30% for testing, using Bhattacharyya measure.

	Ischemia	Benign	Control
Ischemia	1	0	0
Benign	0.43	0.57	0
Control	0	0	1

## Discussion

The tests were carried out using the Euclidean and Bhattacharyya measures so that each sample feature of the five attenuation patterns of ischemia and patterns of the benign and control subjects was classified by the Euclidean distance, while the detection using a much larger proportion of the whole sample population of each class (ischemia/abnormaility, benign and control) was performed by the Bhattacharyya distance. In other words, the Euclidean-distance-based test was designed to carry out the performance of the classification in a detailed way so that the performance of each CT pattern could be analyzed, whereas the Bhattacharyya-distance-based test reflects a practical procedure for realistic applications.

The sensitivity, (the true positive rate or the percentage of diseased subjects who are correctly identified as having the disease) of the results shown in Tables 1, 2, 3, 4 are 98%, 98%, 98% and 82%; respectively. The specificity (true negative rate or the percentage of benign and control subjects who are correctly identified as not having the disease) of the results shown in Tables 1, 2, 3, 4 are 59%, 60%, 58% and 56%; respectively. Based on the results presented in Tables 5, 6, 7, it can be observed that the more the training data were increased, the better the benign was detected (23%, 25% and 57% correction rates obtained for 50%, 60% and 70% training data, respectively); while both ischemia and control feature samples were all correctly classified. The white attenuation pattern was the most misclassified as the control among the five attenuation patterns in three cases with 50%, 60% and 70% of the data used for training. The pattern of the water halo was totally misclassified as the control in the case of applying the leave-one-out method. The fat halo and black patterns appeared to perform the best in all cases. The benign patterns were poorly detected in all cases, which severely affected the specificity. The sensitivity results are much higher than the specificity results in all four cases of testing.

The sensitivity rates of the results shown in Tables 5, 6, 7 are all 100%. The specificity rates shown in Tables 5, 6, 7 are all 50%. The benign was totally misclassified in all three cases, while all samples of the control were correctly classified in all cases. Once again, the sensitivity is as twice as higher than the specificity. Sensitivity has been previously mentioned to be problematic in the assessment of emergency department patients [5]. The high sensitivity reported in this study appears to be promising for the detection of intestinal abnormalities and ischemia.

It has been pointed out that in the assessment of patients with bowel obstruction, an important role of CT findings is to differentiate a simple obstruction from a complicated one, such as a closed-loop or strangulated bowel obstruction; however, the evaluation of this specific CT finding has been based on visual assessment [6]. The proposed feature extraction and classification for automated detection of the disease seems to be promising a potential solution to overcome such problem. Other advanced feature extraction for image analysis such as complexity measures [25] and feature fusion [26] that we have recently reported in similar studies would further improve the current results. Regarding the proposed search for potential regions of interest, although the automated forward search has been shown to work reasonably well and can overcome the demand for an effective image segmentation of the intestines, an accurate image segmentation of the regions of interest would certainly speed up the analysis as well as increase the detection accuracy.

Here we carried out a study on limited cohorts of diseased, benign and control subjects. Future provision of comprehensive CT datasets can be trained to uncover abnormalities in patients with or without symptoms referable to the intestinal tract to better assist the surgeon with the task of urgent diagnosis and operational decision making [12]. It has been realized that intestinal wall morphologic and enhancement abnormalities can be seen with bowel disorders in various ways [12]. This study has not considered the morphology of the CT findings, which can be accommodated using probablistic methods to capture the spatial relationship of the bowel-wall features. Any successful automated detection of ischemia and abnormality is not only helpful to patients admitted to the emergency care, it can also be a useful computer tool to aid the radiologist in developing a systematic approach for determining the specific cause of the intestinal abnormality and ischemia of both small and large intestines.

## Conclusion

CT has been increasingly utilized as an effective, simple and low-cost screening technique for patients with symptoms of the digestive system disease [12]. We have adopted the clinical knowledge about the spectrum of attenuation patterns, including white, gray, water halo sign, fat halo sign, and black to extract these patterns using statistical and geometrical (fractal) methods for the automated noninvasive detection of ischemic bowels. The results presented in terms of sensitivity and specificity are promising for applications to decision making in emergency medicine. When more data become available, an important task is to differentiate these patterns between the benign and ischemia to considerably enhance the diagnostic ability, particularly in the setting of emergency medicine where timely decision making under limited resources is critical. In addition, the proposed approach can be useful for early detection of ischemic bowel disease because people who have been diagnosed with this progressive bowel problem were not aware about having the condition.

## Competing interests

The authors declare that they have no competing interests.

## Authors' contributions

TT conceived the need for automated image analysis of bowel ischemia and drafted the manuscript on the diagnostic performance of CT. TP designed image processing methods and drafted the manuscript on computer algorithms. TF and TS provided insights into the medical aspects of the study. All authors contributed to the revision of the draft and approved the final manuscript.

## Declarations

The publication of this work was supported by FY2013 Strategic Research Grant on "Medical and Biological Image Analyses" funded to TP (Tuan D. Pham).

This article has been published as part of *BioMedical Engineering OnLine *Volume 13 Supplement 1, 2014: Selected articles from the 35th Annual International Conference of the IEEE Engineering in Medicine and Biology Society: Workshop on Current Challenging Image Analysis and Information Processing in Life Sciences. The full contents of the supplement are available online at http://www.biomedical-engineering-online.com/supplements/13/S1
